# Economic modeling of the combined effects of HIV-disease, cholesterol and lipoatrophy based on ACTG 5142 trial data

**DOI:** 10.1186/1478-7547-9-5

**Published:** 2011-05-08

**Authors:** Kit N Simpson, Birgitta Dietz, Robert W Baran, Kevin W Garren, Sharon A Riddler, Menaka Bhor, Richard H Haubrich

**Affiliations:** 1 Medical University of South Carolina, SC, USA; 2 Abbott Laboratories, Abbott Park, IL, USA; 3 Abbott GmbH & Co KG, Ludwigshafen, Germany; 4 University of Pittsburgh, Pittsburgh, PA, USA; 5 University of California, San Diego, CA, USA

**Keywords:** lopinavir/ritonavir, efavirenz, antiretroviral therapy, HIV, AIDS, Markov model, economics

## Abstract

**Background:**

This study examines the cost and consequences of initiating an ARV regimen including Lopinavir/ritonavir (LPV/r) or Efavirenz (EFV), using data from a recent clinical trial in a previously published model of HIV-disease.

**Methods:**

We populated the Markov model of HIV-disease with data from ACTG 5142 study to estimate the economic outcomes of starting ARV therapy with a PI-containing regimen as compared to an NNRTI-containing regimen, given their virologic and immunologic efficacy and effects on cholesterol and lipoatrophy. CNS toxicities and GI tolerability were not included in the model because of their transient nature or low cost remedies, and therefore lack of economic impact. CD4+ T-cell counts and the HIV-1 RNA (viral load) values from the study were used to assign a specific health state (HS) to each patient for each quarter year. The resulting frequencies used as "raw" data directly into the model obviate the reliance on statistical tests, and allow the model to reflect actual patient behavior in the clinical trial. An HS just below the last observed HS was used to replace a missing value.

**Results:**

The modeled estimates (undiscounted) for the LPV/r-based regimen resulted in 1.41 quality-adjusted life months (QALMs) gained over a lifetime compared to the EFV-based regimen. The LPV/r-based regimen incurred $7,458 (1.8%) greater cost over a lifetime due to differences in drug costs and survival. The incremental cost effectiveness ratio using the discounted cost and QALYs was $88,829/QALY. Most of the higher costs accrue before the 7th year of treatment and were offset by subsequent savings. The estimates are highly sensitive to the effect of lipoatrophy on Health-related Quality of Life (HRQOL), but not to the effect of cholesterol levels.

**Conclusions:**

The cost effectiveness of ARV regimens may be strongly affected by enduring AEs, such as lipoatrophy. It is important to consider specific AE effects from all drugs in a regimen when ARVs are compared.

**Trial registration:**

(ClinicalTrials.gov number, NCT00050895http://[ClinicalTrials.gov]).

## Background

The use of combination antiretroviral therapy (ART) has led to a well-documented trend of declining AIDS-related morbidity and mortality among HIV-positive patients [1-3]. Treatment strategies for HIV/AIDS have changed over time [4-6] as therapies have evolved to become more convenient and tolerable. For treatment naïve patients, current DHHS and other guidelines recommend regimens with two nucleoside reverse transcriptase inhibitors (NRTIs) and either a protease inhibitor (PI), an integrase strand transfer inhibitor (INSTI) or a non-nucleoside reverse transcriptase inhibitor (NNRTI) [7,8]. Both NNRTI- and PI-based regimens result in suppression of HIV RNA levels and CD4+ T-cell increases in a large majority of patients [9-13]. The use of ritonavir-boosted PIs have led to improved virological suppression compared to non-ritonavir PI regimens, as detailed in clinical trials [[14,15], and [16]] and cohort studies [17], as well as improved clinical outcomes in observational cohort studies [18].

Head-to-head randomized clinical trials are accepted as the most powerful tool for assessing the effectiveness of medical interventions. The AIDS Clinical Trials Group (ACTG) 5142 study was a large, randomized, phase III trial that was designed to compare the efficacy of 2 recommended first-line regimens-an NNRTI-based regimen consisting of efavirenz (EFV) plus 2 NRTIs and a PI-based regimen consisting of lopinavir/ritonavir (LPV/r) plus 2 NRTIs. In terms of virologic outcomes, the EFV-based regimen was more effective with significantly higher rates of virologic suppression and longer time to virologic failure than LPV/r plus 2 NRTIs [12].

In the ACTG 5142 study, although patients were less likely to experience virologic failure with the EFV-based regimens, those who did fail on EFV-based regimen (26%) were significantly (P < 0.001) more likely to have mutations associated with resistance to two drug classes than those who failed after receiving LPV/r plus 2 NRTIs(1%) [12]. For the two study arms used in modeling analysis, the resistance was 9% for the EFV-based regimen and 6% for the LPV-based arm.

Previous retrospective and cross-study comparisons have suggested that CD4+ T-cell recovery is better with PI regimens than with NNRTI-based regimens [19,20]. In ACTG5142 patients had a significantly (p = 0.01) greater CD4+ T-cell count increase from baseline to week 96 on the LPV/r-containing regimen (287 cells per cubic millimeter, as compared to the EFV-containing regimen (230 cells per cubic millimeter) [12].

Lipoatrophy (fat loss usually seen in the face, arms, legs and buttock area) remains among the most devastating, and even stigmatizing, side effects of antiretroviral medications. Lipoatrophy is associated with a negative impact on the Health Related Quality of Life (HRQOL) in HIV-infected individuals [21]. The incidence of lipoatrophy can be attributed to use of thymidine analogues as NRTIs. In the ACTG 5142 the NRTI of choice was Zidovudine (ZDV) 42%, stavudine (d4T XR ) 24%, and Tenofovir (TDF 34%). By week 96 of the ACTG 5142 trial the DEXA defined lipoatrophy in the EFV + NRTI (32%) or LPV+NRTI (17%) arms was predominantly seen in the d4T- or ZDV-containing regimens; there was no significant difference (p > 0.5) in lipoatrophy between TDF- containing (LPV-TDF 6% and EFV-TDF: 12%) and NRTI-sparing regimens (9%). Overall EFV was associated with a 2.7 times increased risk of developing lipoatrophy (which was defined as a loss of >20% in fat (ACTG definition)) when used with 2 NRTIs compared to LPV/r when used with 2 NRTIs [22].

Under these premises, the LPV/r-containing ARV regimen is expected to be more beneficial in terms of genetic barrier to resistance and also a reduced propensity to lipoatrophy compared to the EFV-based regimen. These attributes potentially increase the value of LPV/r in terms of health and economic outcomes. However there was a major trade-off between the regimens: Failure was less common with EFV plus 2 NRTIs, but the impact of failure was greater in terms of increased rate of resistance. This study examines the expected long term cost and consequences of initiating an ARV regimen including LPV/r or EFV, using data from two of the three arms in the ACTG 5142 clinical trial that compared EFV plus two NRTIs and LPV plus 2 NRTIS. These data were used as parameters in a previously published Markov model for HIV-disease which is described below.

## Methods

### Study Population

The study population consisted of HIV-1-infected male and female patients at least 13 years of age who had not received previous ART and participated in the ACTG 5142 study. Data from the 2 NRTI-containing arms with LPV or EFV were used for this analysis.

### Study Design

A Markov model of HIV-disease [23,24,21] was populated with data (on viral load, CD4+ T-cell count, treatment-emergent resistance, treatment-emergent lipoatrophy (measured by DEXA scan) and health-related quality of life (HRQOL) from the ACTG 5142 study to estimate the economic outcomes of starting ARV therapy with a PI-containing regimen as compared to an NNRTI-containing regimen, given their virologic and immunologic efficacy and effects on cholesterol and lipoatrophy. The effects of CNS toxicities were not included in the model because of their often transient nature, and the effects of diarrhea were not included in the model because of short duration which decreased overtime, the low cost remedies used in management, and lack of significant effect on patients' quality of life measure (p = .0818) in the trial data and hence lack of economic impact on the model results.

### Model Structure and Health States

**Figure 1:**
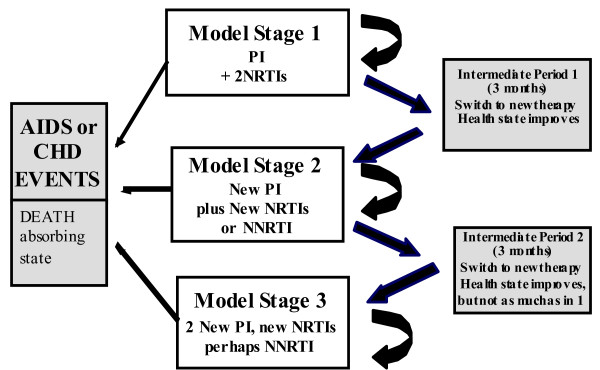
**Model Structure**.

The base model structure used in this study is depicted in Figure 1. This model has been used previously to estimate economic outcomes for LPV/r, atazanavir, and tipranavir [24,21] and its structure, assumptions and predictive validity has been published elsewhere [23].

The main efficacy measures were based on the observed CD4+ T-cell counts and the viral load (VL) values from the study. The baseline value for the CD4+ T-cell count was used, while the last recorded (entry) VL value was used to define a patient's health state (HS) at baseline. The resulting frequencies were used as "raw" data and populated directly into the model. This allows the model to be based on the actual behavior of the CD4+ T-cell counts and VL recorded in the clinical trial instead of using a mean or median estimate as an input.

The clinical trial period after randomization (96 weeks) was divided into 8 quarters, and a HS was assigned to each patient for each quarter based on the recorded CD4+ T-cell counts and viral load values. The mean quarter value for the CD4+ T-cell count, and the last recorded VL value in the quarter were used to define a patient's HS. The percent distribution for the model HS for the first four quarters for each regimen was used to populate the model HSs for those quarters. Patients without observations for a quarter were treated as failures and assigned to an HS just below the last observed HS.

### Drop-out Rate

There were no differences between the LPV/r and EFV regimens in the distribution of dropouts by quarter in the data used to populate the model (p = 0.2801), nor did the distribution of dropouts for the two regimens differ by the last HS occupied (p = 0.8674).

### Failure Rate

The Markov model has in the past used a transition matrix that was based on data from 1999 and 2000 for antiretroviral-naïve patients. However, recent data presented for ARV-experienced patients indicate that average failure rates were reduced by about 50 percent between 2000-2001 and 2005 [25]. This finding required that the failure rates for the study patients after the fourth quarter be compared to the failure rates assumed in the model's transition matrix. To do this the observations after 52 weeks were classified by model HS for each subsequent quarter in the manner described above for the early quarters. These data were then used to estimate the failure rates (transitions) expected after the end of the fourth quarter. While there were no significant differences in the failure rates for the two regimens (p = 0.3691), the study failure rates were somewhat improved over the rates used in the original model transition matrix. Thus, the model transition matrix was updated using the observed study failure rates for all health states that had at least 20 transition observations. The percent of patients in HS1, HS3, HS5 and HS8 (the undetectable VL health states), which had VL below 50 copies/ml for the two treatment regimens, were also examined. There were no differences in the proportion of patients in HSs with undetectable VL whose VL was below 50 copies/ml for the two regimens in quarter 4 (p = 0.1021) and in quarter 8 (p = 0.1028). Thus a transition matrix, which was updated using the pooled study data, was used to estimate the regimen's progression in the model after end of the initial four quarters, making the failure rates used in the model identical for the EFV and LPV/r regimen for the time period after the end of the study data.

### Health Related Quality of Life Adjustment

The Markov model has in the past used utility weights that were extracted from pooled EuroQoL (EQ5D) data from about 21,000 responses from patients enrolled in a large number of early ARV studies [26]. However, there is anecdotal evidence that today's ARV regimens may result in a different level of health related quality of life than older ARV regimens. The ACTG 5142 trial data included a generalized health-related quality of life question which could be converted to utility weights using a simple linear transformation where the utility u = 0.44 V + 0.49 (V = the visual analog score given by the patient) as reported by Mrus and colleagues (2003) [27]. The resulting utility weights for the model health states were generally decreasing as the CD4 + T-Cells and VL-defined health states worsened. The new utility values were used in the Base Estimate, and the effects of using the original model utility weights tested in the sensitivity analysis. The original model health state utility values and the values that are based on the ACTG trial data are provided in Table 1. The ACTG 5142 utility weights exhibit less monotonicity, probably because of a much smaller sample size for each of the health states.

**Table 1:** Original Model Utility Weights and Utility Weights Based on ACTG5142 Data

Health State	Original Model Utility Weights	ACTG5142 Utility Weights (SD)
HS 1	0.954	0.849 (.068)
HS 2	0.938	0.851 (.052)
HS 3	0.934	0.852 (.062)
HS 4	0.931	0.825 (.066)
HS 5	0.929	0.839 (.072)
HS 6	0.931	0.819 (.080)
HS 7	0.933	0.820 (.087)
HS 8	0.863	0.829 (.077)
HS 9	0.865	0.830 (.092)
HS 10	0.826	0.722 (.109)
HS 11	0.876	0.783 (.099)
HS 12	0.781	0.792 (.088)

### Lipoatrophy Sub-Model

The original model did not take the development of lipoatrophy into account when estimating the health related quality of life (HRQOL) estimated from each of the treatment regimens. However, Haubrich and colleagues (2007) [22] reported lipoatrophy-defined by DEXA scan at 96 weeks (LPV/r = 17%; EFV = 32%). We used the percent of patients with 20% loss of limb fat by DEXA measurement, as defined by the study protocol as the basis for estimating the differences in the proportion expected to develop lipoatrophy over time for the two regimens. Assumptions related to the effects of the rate of lipoatrophy were tested in the sensitivity analysis. The economic effect of lipoatrophy was assumed to be limited to 10% of individuals with the condition, and to develop slowly over a five year period. The effect of lipoatrophy on HRQOL was estimated using a utility decrement approach based on the average decrement observed across all individuals in the study. The ACTG5142 study collected data on participants' reported body changes due to lipoatrophy that included three questions. Using those data, we calculated decrements in utilities due to lipoatrophy for the model. The questions of interest were related to fat redistribution in the face, buttocks, arms, and legs. Patients who answered 'yes' to these questions reported significantly lower utility weights than patients who answered 'no' to the re-distribution of body fat. Since there was a difference between treatments in the proportion of patients who developed lipoatrophy based on DEXA scan (not including facial lipoatrophy) in the ACTG 5142 study, we constructed a sub-model that assigned a decrement of 0.05 utility due to the effects of lipoatrophy on HRQOL. The results of the analysis of the utility values for patients with and without evidence of lipoatrophy are provided in Table 2.

**Table 2:** Effect of Lipoatrophy on Utility Weights

Lipoatrophy Symptoms	Yes (SD)	No (SD)	P value*
Have your cheeks sunken?	0.811 (.073)	0.846 (.071)	<0.0001
Have you lost fat in the butt?	0.813 (.079)	0.848 (.069)	<0.0001
Have you lost fat in your arms and legs?	0.815 (.080)	0.848 (.069)	<0.0001
Mean utility decrement controlling for HS	-0.052		

Lipoatrophy may increase cost of care for some patients. Some patients will seek treatment for this condition. The model assumes that 1.7 and 3.2 percent (LPV/r and EFV groups respectively) of patients seek treatment for lipoatrophy. Treatment consists of 30 ml Poly-lactic acid injections every 3 years at a cost of $4,190 [28] per treatment. In the model this cost is assigned as $35 per quarter over the time with lipoatrophy. This assumption allows the model to accommodate the fact that clinical lipoatrophy developed slowly over time, and that only a small fraction of patients seek treatment for the condition.

### Cost Data Sources

Cost per AIDS event is based on average costs calculated from the analysis of U.S. Medicaid payment and hospital all-payer discharge data for patients with AIDS diagnoses. Cost resulting from added risk of coronary heart disease (CHD) due to increased total cholesterol values are estimated based on hospitalization cost data for patients with a myocardial infarction (MI) diagnosis. Average cost per AIDS event is $31,881 (range $1,093 for cervical cancer to $214,280 for CMV retinitis) [29]. Cost per CHD event is $25,423 based on average costs for hospital admissions for MI patients in the US in 2005 [30]. Cost of lipid-lowering therapy is assumed to be $2.68 per day, and this value is used for the remaining lifetime. The ART drug costs are based on the US daily average wholesale price [31]. These are $26.54 for LPV/r tablets, $16.65 for EFV, $26.19 for the NRTI backbone, $30.07 for darunavir, $68.07 for enfuvirtide, and $14.75 for etravirine. All other model costs are reported as the 2007 present value in US currency. Costs and outcomes are discounted by 3 percent for the calculation of the incremental cost effectiveness and cost utility ratios. The perspective of the analysis is that of the government/third party payer, and does not include indirect costs in the model cost estimates. These model input factors are summarized in Table 3.

**Table 3:** Cost Parameters Used in the Base-Model and Sources of Costs

Description	Unit Cost	Source
Mean cost per AIDS event	$31,881	SC Medicaid population [29]
Mean cost per MI event	$25,423	SC Medicaid population
Cost per lipoatrophy treatment	$4,190	Hornberger [28]
Cost per monitoring visit	$334	SC Medicaid population
Cost of switching ARV regimen	$334	SC Medicaid population
Lipid-lowering drugs, cost per day	$2.68	AWP Red Book 2007 [31]
LPV/r cost per day	$26.54	AWP Red Book 2007
EFV cost per day	$16.65	AWP Red Book 2007
NRTI backbone, cost per day	$26.19	AWP Red Book 2007
Darunavir cost per day	$30.07	AWP Red Book 2007
Etravirine cost per day	$14.75	AWP Red Book 2007
Enfuvirtide cost per day	$68.07	AWP Red Book 2007

### Other Assumptions

Cholesterol levels were assumed to be equal for the two regimens based on the published study report [12].

The 2^nd ^regimen for patient who received LPV/r initially was assumed to be EFV-based, (and vice versa) based on the stipulation in the trial protocol, the third regimen in the model was assumed to be based on Darunavir. After 96 weeks in the clinical trial, 19 percent of patients with virologic failure on LPV/r and 30 percent of patients on EFV were reported to have NRTI resistance [32]. The overall study rate of resistant mutations observed were 9 percent for the EFV regimen and 6 percent for LPV/r regimen.

In the resistance data for all virological failures in the trial, there were no cases where a second ARV regimen with three fully active drugs could not be constructed. Thus, the resistance rates were used only for estimating the cost of the third regimen. It was assumed that patients with any virus mutation that was resistant after the first regimen (EFV = 30% and LPV/r = 19% based on the trial resistance data for the proportion of patients with virologic failure who had NRTI resistance) would require a more complex drug regimen after a second failure. The effects of 6 and 9 percent resistance, 6 percent resistance for both regimens, and no resistance effects on the third regimen are modeled in the sensitivity analyses. The base model assumption was that 75 percent of patients with resistant virus would receive etravirine and that 25 percent would receive enfuvirtide as part of their third regimen. This reflects the current guideline recommendation that a new regimen should have at least 2 and preferably 3 active drugs, if possible.

### Patient Distribution at Baseline

We compared the differences in the distribution of patients among the eight possible model HS (HS with undetectable VL are not possible at baseline) for the LPV/r arm and the efavirenz (EFV) arm using a Chi square test (Table 4). This comparison is needed because randomization does not always assure a comparable distribution of surrogate markers across a Markov model's HS at baseline. We found a significant difference in the distribution of patients among the baseline HS, with EFV patients being distributed more towards the extreme HS, and LPV/r patients distributed more in the middle HS (p = 0.0301).

**Table 4:** Patient Distribution Between the Model Health States at Baseline

Base Health State	CD4 Range	VL Range	EFV Percent	LPV/r Percent	Difference %*
2	>500	>400	6.4	2.8	3.6
4	350-499	>400	14.8	11.4	3.4
6	200-349	400-10,000	8.8	8.7	0.1
7	200-349	>10,000	18.8	23.2	-4.4
9	50-199	400-10,000	3.6	2.4	1.2
10	50-199	10,001-100,000	10.0	16.9	-6.9
11	50-199	>100,000	11.2	15.4	-4.2
12	<50	any	26.4	19.3	7.1

*Chi-Square 15.5; p = 0.0301

This significant difference in the distribution of patients among the HS at baseline required an analysis to estimate the effect of this potential bias on the cost effectiveness of the two study regimens. To examine this effect the maximum observations within each baseline HS were randomly selected for each regimen and the data from this smaller cohort were used in a sensitivity analysis. The baseline distribution between health states for this sub-population is provided in Table 5.

**Table 5:** Baseline Distribution among the Model Health States after Random Selection of Patients (n = 213 per arm)

Base Health State	CD4 Range	VL Range	EFV Number of Patients	LPV/r Number of Patients
2	>500	>400	7	7
4	350-499	>400	29	29
6	200-349	400-10,000	22	22
7	200-349	>10,000	47	47
9	50-199	400-10,000	6	6
10	50-199	10,001-100,000	25	25
11	50-199	>100,000	28	28
12	<50	Any	49	49

## Results

The estimates for the Base Model are provided in Table 6. The modeled estimates (undiscounted) for the LPV/r-based regimen resulted in 1.41 quality-adjusted life months (QALMs) gained over a lifetime compared to the EFV-based regimen. The LPV/r-based regimen incurred $7,458 (1.8%) greater cost over a lifetime due to differences in drug costs and survival. The incremental cost effectiveness ratio using the discounted cost and QALYs is $88,829/QALY. Based on the Budget Impact model (Table 6) there was a 2.7% increase in ARV budget lifetime (undiscounted) costs for patients starting on LPV/r-based regimen as compared to patients who started on EFV based regimen. The estimates for the model using a random selection of patients that are equally distributed among the health states at baseline are provided in Table 7.

**Table 6:** Cost, Consequences per 100 Patients, and Cost Effectiveness of Using an Initial Antirethroviral Regimen of LPV/r Followed by Efavirenz

Variable Estimated	LPV/r	EFV	Difference	ICER
Undiscounted QALYs	1,163	1,151	11.7	
**QALY months gained per person**			**1.41 months**	
QALYs discounted	944	935	9.388	
Costs discounted	$32,365,777	$31,531,823	$833,953	
**Cost per QALY**				$88,829/QALY*
5 year mean total cost/patient undiscounted	$115,219	$103,226	$11,994	
10 year mean total cost/patient undiscounted	$221,428	$211,121	$10,307	
Lifetime mean total cost/patient undiscounted	$413,767	$406,309	$7,458	1.8% increase for LPV/r
**ANTIRETROVIRAL**	**BUDGET**	**IMPACT**		
	**LPV/r**	**EFV**	**Difference**	**Percent Increase**
5 year cost of ARV drugs per patient (undiscounted)	$90,336	$78,536	$11,800	
10 year cost of ARV drugs per patient (undiscounted)	$172,421	$162,160	$10,261	
**Percent Lifetime ARV budget increase estimated for using LPV/r first, per patient (undiscounted)**	**$279,697**	**$272,289**	**$7,408**	**2.7%**

* Errors due to rounding

**Table 7:** Health Outcomes and Cost Effectiveness for the Base Model and the Baseline-adjusted Model

Variable Estimated	Base Model	Adjusted Baseline Model	"Old" Utility Values Model
QALY months gained per person	1.41 months	1.04 months	1.44 months
Cost per QALY for LPV/r	$88,829/QALY	$117,234/QALY	$86,256/QALY

### Sensitivity Analysis

**Figure 2:**
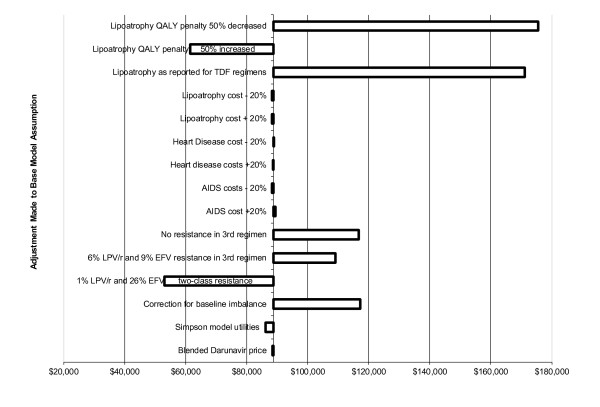
**Effects of Sensitivity Analysis on the Incremental Cost Effectiveness Ratio Estimates for the Model**.

The results of the sensitivity analysis of the effects of key model assumptions on the Incremental Cost Effectiveness Ratio (ICER) are presented in Table 8 and Figure 2.

**Table 8:** Base Model Estimate and Sensitivity Analysis of the Effects of Key Model Assumption on the Incremental Cost Effectiveness Ratio

Changes of Assumptions in the Model	Cost per QALY
**Base estimate**	**$88,829**
This model assumes that 19% of patients who fail the LPV/r with ANY resistance and the 30% who fail EFV with ANY resistance will have Etravirine added to Darunavir as their 3^rd ^regimen	$98,581
As above but using darunavir blended price* of $43.85 per day in 3^rd ^regimen	$98,210
Base model but using the utility values from the published Simpson model	$95,432
This model assumes that the 1% of patients who fail the LPV/r with 2 class resistance and the 26% who fail EFV with 2 class resistance will have Etravirine added to Darunavir as their 3^rd ^regimen	$53,095
This model assumes that 6% of patients fail the LPV/r with NRTI resistance and 9% fail EFV with NRTI resistance, and that these patients will have Etravirine added to Darunavir as their 3^rd ^regimen	$116,797
This model assumes that there is no effect of choice of first regimen on the cost of the 3^rd ^treatment due to resistance	$116,774
Change AIDS event cost +20% or -20%	$99,238 and 97,924
Change heart disease cost +20% or -20%	$98,583 and $98,579
Change Lipoatrophy cost +20% or - 20%	$98,108 and $99,054
No cost of treating lipoatrophy	$91,226
Lipoatrophy rates 6% and 12% as observed in the TDF sub-groups	$171,187
Change Lipoatrophy QALY to "+50% and -50%" (from -.052 in base model to -.026 or -.078)	$175,538 and $68,535

* Blended price is average selling price (ASP) across all the channels of market

## Discussion

This study uses a decision-analysis modeling approach with the model inputs derived from patient-level clinical trial data to compare the expected long term economic and HRQOL consequences of initiating ART therapy with an NNRTI-based vs. a PI-based regimen for treatment-naive patients. The resulting modeling estimates provide information on the importance of judging clinical trial results for ARV regimens on more than simply the VL suppression at 48 weeks under intent-to-treat analytical assumptions.

The model estimated an increase of 1.41 months per patient of quality adjusted survival for the PI-based cohort. This difference was mainly due to the higher rate of lipoatrophy in the NNRTI-arm of the study. It is not the cost of treating lipoatrophy that appears to be the most important factor in the model. When we changed the cost of treating this AE the predicted ICER increases minimally from $88,829/QALY to $91,226/QALY. If the cost of the EFV regimen increases by $4.60/day then the LPV/r regimen becomes dominant. However, when we assume a 50 percent reduction in the HRQOL weight associated with lipoatrophy the ICER increases from $88,829/QALY in the base model, to $175,538/QALY (see figure 2). Thus, the effect of lipoatrophy on patients' quality of life is a much more important variable than is the cost of treating this condition. This is an important issue, since the differential rate of lipoatrophy reported in the study may be partially due to the NRTI backbone combinations used in ACTG5142. Since the study evaluated NRTI-backbone regimens that are no longer recommended by the guidelines [7] for initial ARV treatment and which are currently not used in clinical practice, the effect on the ICER of AEs that may be more strongly associated with specific NRTI drugs should be noted.

The results of this study makes it clear that short and medium time cost savings resulting for a choice of ARV therapy are not synonymous with cost effectiveness when lifetime impacts are considered. The model estimated mean cost savings of $11,994, $10,307, and $7,458 per patient at years 5, 10, and lifetime, respectively for the NNRTI cohort. The incremental cost effectiveness ratio (ICER) for the LPV/r regimen in the base model was $88,829/QALY gained, which is considered cost effective for the US under the WHO criteria [33].

However, the lifetime incremental cost effectiveness ratios (ICER) for the two regimens varied greatly. The ICER for the LPV/r regimen depends on the cost assumptions used in the model, the effects of different model assumptions with regards to the second and third ARV regimens to which the population was switched once the initial regimen failed, and the utility values associated with lipoatrophy. Assumptions varied in the sensitivity analyses resulted in varying the ICER estimates between $68,535 and $175,538. The adjustment of the population at baseline that was introduced to examine the effects of uneven distribution of patients among the model health states at baseline changed the ICER to $117,234/QALY. However, changing the utility weight for patients who experienced lipoatrophy resulted in ICERs between $68,535/QALY and $175,538/QALY for the LPV/r group, depending on the assumptions about the utility weight (Table 8). The ICER increases to $171,187/QALY when we assume that the rates of lipoatrophy are 6 and 12 percent (LPV/r and EFV respectively) as were reported for the TDF subgroup in the trial. These findings illustrate the fact that when economic, quality of life and patient preferences are all considered, then there is probably no "best" regimen for all patients. The volatility of the ICER when assumptions for AE rates and the risk of developing resistance to the third regimen are changed indicate that the value generated by a specific ARV regimen choice may be greatly affected by how much the adverse effects associated with a regimen affect a patient's HRQOL, and the level of risk of the virus becoming resistant to future regimens.

This decision analysis study used a Markov model for estimation, and any modeling result is only as good as the ability of the model's structure to capture the essential aspects of the disease and treatment process. We have used a peer-reviewed and previously published model [24] in this analysis to minimize any bias which could be caused by a poorly structured model. However, the validity of an estimate from a model is also highly dependent on the validity of the parameters used in the model. We have used simple frequencies calculated from the "raw" data for the first four quarters of the clinical trial of the two drug regimens [12] to populate the model. This approach, while simplistic, has several advantages: 1) it reflects the actual behavior of the data in the study, including the correlation between variables; and 2) it is simple to understand, and not dependent on statistical tests of significance which are affected by sample size and the innate variation in measurements. We have tested the effects of the variations in the data and of the assumptions made in the model for progression after the end of the clinical trial by performing sensitivity analyses that use different assumptions and utility weights. This approach helps in the identification of the most important factors that may affect the modeling estimates.

Thus, the modeling estimates capture many of the major variations in long term cost and health related quality of life that may be expected from the cohorts of patients that contributed to the trial data. The model is limited in that CNS and gastrointestinal side effects (which can sometimes be chronic) are not included in the model. Randomized clinical trial results are the gold standard for defining safety and efficacy of therapy, but are limited to the relatively short duration of the study in comparison with life-long treatment currently needed for HIV- infection. This study illustrates the fact that costs, health related quality of life, adverse events, and the effect of resistance on the mixture of drugs in subsequent regimens interact and may affect long term cost and consequences.

## Conclusions

Based on the assumptions made in the model, it appears that the choice of an initial ART regimen for treatment-naive patients should consider how adverse an individual patient is to specific side effects of a regimen, in addition to more commonly recognized issues, such as the rate of adverse effects, AIDS-related events and opportunistic infections that warrant highly expensive treatments, as well as the ART-regimen's acquisition cost, expected effects on viral load suppression, CD4 + T-cell increase, and resistance induced to subsequent regimens.

## Competing interests

KS was the principal investigator on a grant by Abbott to MUSC to perform the study. BD, RB KG and MB are Abbott employees. SR and RH have no competing interests.

## Authors' contributions

KS analyzed the ACTG 5142 data, conceptualized and programmed the economic model, and lead the writing of the manuscript. BD and RB provided model cost input data and collaborated on writing the manuscript. MB drafted parts of the manuscript. KG SR and RH participated in the design of the study, lead the interpretation of the ACTG 5142 data and collaborated on writing the manuscript. All authors read and approved the final manuscript.
